# Reduction in Standard Cancer Screening in 2020 throughout the U.S.

**DOI:** 10.3390/cancers13235918

**Published:** 2021-11-25

**Authors:** Leslie K. Dennis, Chiu-Hsieh Hsu, Amanda K. Arrington

**Affiliations:** 1 Department of Epidemiology and Biostatistics, Mel and Enid Zuckerman College of Public Health, University of Arizona, Tucson, AZ 85724, USA; pchhsu@arizona.edu; 2 University of Arizona Cancer Center, Tucson, AZ 85724, USA; akarrington@houstonmethodist.org; 3 Environment, Exposure Science and Risk Assessment Center, Mel and Enid Zuckerman College of Public Health, University of Arizona, Tucson, AZ 85724, USA; 4 Department of Surgery, Houston Methodist Hospital, Houston, TX 77030, USA

**Keywords:** cancer screening, COVID-19, colonoscopy, disparities, mammogram, pap test, sigmoidoscopy

## Abstract

**Simple Summary:**

The COVID-19 pandemic has had a significant impact on health care including cancer screening. We examined 2020 compared to the 2014–2019 cancer screening percentages in the United States (US) based on a national survey. We saw overall decreases in screening mammograms, pap tests, and sigmoidoscopy/colonoscopy. Most decreases were higher among American Indian/Alaskan Natives, Hispanics, and multiracial participants, but decreases in pap test were also high among African-Americans/Blacks. As the pandemic expanded into 2021, cancer screening reduction is expected to be higher, increasing cancer disparities.

**Abstract:**

Cancer screening is an important way to reduce the burden of cancer. The COVID-19 pandemic created delays in screening with the potential to increase cancer disparities in the United States (U.S.). Data from the 2014–2020 Behavioral Risk Factor Surveillance System (BRFSS) survey were analyzed to estimate the percentages of adults who reported cancer screening in the last 12 months consistent with the U.S. Preventive Services Task Force (USPSTF) recommendation for cervical (ages 21–65), breast (ages 50–74), and colorectal cancer (ages 50–75) prior to the pandemic. Cancer screening percentages for 2020 (April–December excluding January–March) were compared to screening percentages for 2014–2019 to begin to look at the impact of the COVID-19 pandemic. Screening percentages for 2020 were decreased from those for 2014–2019 including several underserved racial groups. Decreases in mammography and colonoscopy or sigmoidoscopy were higher among American Indian/Alaskan Natives, Hispanics, and multiracial participants, but decreases in pap test were also highest among Hispanics, Whites, Asians, and African-Americans/Blacks. Decreases in mammograms among women ages 40–49 were also seen. As the 2020 comparison is conservative, the 2021 decreases in cancer screening are expected to be much greater and are likely to increase cancer disparities substantially.

## 1. Introduction

Cancer is the second leading cause of death in the United States (U.S.), just behind heart disease in 2019. Cancer screening has been proven to be an effective preventive measure that can reduce cancer incidence and mortality [1]. COVID-19 (Coronavirus disease 2019) is a severe acute respiratory syndrome coronavirus 2 (SARS-CoV-2) that expanded into a pandemic in March 2020, severely affecting our society, which has induced unprecedented ramifications [2,3]. Due to the COVID-19 pandemic, many medical centers have reported that 2020 screenings were canceled or postponed [4,5]. Additionally, the COVID-19 pandemic decreased the patients’ willingness to go to medical clinics (in general) and to undergo screening due to the fear of contracting COVID-19 [6]. The complete repercussions of the COVID-19 pandemic on cancer screening, rates, treatment, and mortality are unknown. Estimated cumulative excess cancer-specific mortality compared to undisrupted screening suggests a possible 2-fold increase for breast cancer and more than twice the mortality of colorectal cancer [7]; based on cancer-specific mortality in the U.S. [8], this could lead to over 90,000 additional cancer specific deaths over 2020–2030. There is vital need for studies investigating how the pandemic is affecting the uptake of cancer screening, especially among the medically underserved [9], as reduced uptake could lead to later stage diagnosis and increased mortality.

Cancer disparities have existed in the U.S. for many decades. Socioeconomic related risk factors for cancer are related to social determinants of health: factors that are shaped by the distribution of money, power, and resources. Cancer disparities, particularly for CRC, are also affected by significant differences in social determinants of health such as unhealthy diet, food insecurities, and sedentary lifestyle; limited access to risk-reducing behaviors such as screening, chemoprevention, and follow-up of abnormal test results; or lack of transportation or time off from work to access high-quality treatment resources [9]. Given this, incidence rates for CRC and cervical cancer are higher in historically disadvantaged groups [8,10]. For breast, cervical, and colorectal cancers, structural disparities have had clear effects on screening for the early detection of cancers and thus mortality. Blacks have higher rates of mortality in the U.S. for breast, cervical, colorectal, and prostate cancers [8]. Hispanics have higher mortalities than non-Hispanic Whites for cervical cancer [11]. Depending on the reporting source, American Indians/Alaska Natives have higher mortality rates for cervical, colorectal, and prostate cancer [8]. Minorities and medically underserved populations showed lower uptake of cancer screening than non-Hispanic Whites [9,12]. However, expansions in Medicaid and many screening programs for low-income adults has increased screening, particularly among Hispanics [13,14]. Prior analyses of BRFSS data have shown 6–10% higher prevalence of meeting screening guidelines for breast, cervical, and colorectal cancers among non-Hispanic Black women than among non-Hispanic White women [12], further suggesting an effect of expanded screening programs. However, the onset of the COVID-19 pandemic is believed to have caused many people to not have routine checks-ups and screening [4,15,16]. It is expected that many people put off cancer screening 3–18 months due to COVID-19 and so cancer disparities will be increased.

The purpose of these analyses was to examine cancer screening in the U.S. based on the annual Behavioral Risk Factor Surveillance System (BRFSS) survey. Preventive screening questions included asking about ever having a mammogram for breast cancer (women only), a pap test for cervical cancer (women only), and ever had sigmoidoscopy, or colonoscopy to detect colorectal cancer (CRC). The BRFSS further asked for these cancer screening procedures how long it had been since their last screening. We compared the BRFSS participants’ screening status in the past 12 months by race/ethnicity by comparing 2014–2019 to 2020 to see whether a reduction in screening was seen in 2020.

## 2. Materials and Methods

### 2.1. The Behavioral Risk Factor Surveillance System (BRFSS)

The Behavioral Risk Factor Surveillance System (BRFSS) is a premier system of health-related telephone surveys that collect state data about U.S. residents regarding health-related behavior, chronic health conditions, and the use of preventive services. BRFSS collects data from all 50 states as well as the district of Columbia and three U.S. territories. It conducts more than 400,000 adult interviews per year. The survey was redesigned to include rotating fixed core and rotating core questions and up to five emerging core questions. In 2014, the survey consisted of core questions, optional modules, and state added questions. The new methodology uses ranking and iterative proportional fitting but maintains representativeness, coverage, and validity of the BRFSS data. Random-digit-dialing of households is used for landlines and cellphones throughout the U.S. (https://www.cdc.gov/brfss/ accessed on 21 November 2021). The preventive services questions included questions about cancer screening.

Screening rates were core questions asked by all states and the District of Columbia in even years (2014, 2016, 2018, and 2020). For odd years, only some states asked the screening questions, thus the 2014–2019 data were examined together. New Jersey did not participate in the BRFSS in 2019. Since screening rates for the Virgin Islands were only asked in 2016, we excluded the three U.S. territories. Colorectal cancer screening for 2014–2019 was only asked for participants aged 50+, but for 2020, it was asked for ages 45+. For consistency to compare screening percentages for colorectal cancer, we restricted this to ages between 50 and 75 in the analyses based on the U.S. Preventive Task Force recommendations. Breast cancer screening was restricted to women aged 50–74 and cervical cancer screening to 21–65 based on the U.S. Preventive Task Force recommendations [12].

The BRFSS has standard screening questions including ever having a mammogram, pap test, sigmoidoscopy, or colonoscopy, along with time since having each of these tests (anytime less than 12 months ago, one year but less than two years ago; two years but less than three years ago; three years but less than five years ago; or five or more years ago). However, since sigmoidoscopy and colonoscopy exams are recommended less frequently, the upper categories for time since last test were longer (5 years but less than 10 years ago, or 10 or more years ago). Mammography (ever had a mammogram and how long since your last mammogram) was asked along with having had a pap test for cervical cancer.

CRC screenings were asked in different modules, so they could have been asked by different states in odd years than the breast and cervical cancer screening. CRC screening evaluation and analysis is complex. There are multiple acceptable forms of CRC screening: guaiac-based fecal occult blood testing (FOBT), fecal immunohistochemical testing (FIT), multitarget stool DNA test (Cologuard), virtual colonoscopy (computed tomography (CT), colonography), flexible sigmoidoscopy, and colonoscopy. Sigmoidoscopy and colonoscopy have been standard screening for a long time, whereas, virtual colonoscopy is very new and not assessed via BRFSS. Blood stool tests are recently becoming popular, but less than 10% of participants reported these tests; this proportion has been increasing since 2014. Thus, blood stool tests were not compared pre- and during COVID-19. The definitions of and the questions referring to colonoscopy and sigmoidoscopy varied pre-2020 and in 2020.

### 2.2. BRFSS Data Preparation

Data from the BRFSS survey designed by the Centers for Disease Control and Prevention (CDC) were downloaded for each year of the survey from 2014–2020. For each year, the BRFSS codebook was checked for changes in coding. Data were read into SAS using the BRFSS provided SAS programs for each year. As needed, data from year to year were compared for consistent coding of sex, race, and Hispanic ethnicity. Sex was coded as a different variable in 2014–2017, 2018, 2019–2020, so was checked and coded into one variable. The datasets for each year were large, so after merging by year, we only kept variables for demographics (age, sex, race, Hispanic, education, income) and cancer screening variables (breast, cervical, and colorectal cancers) for analyses.

To evaluate changes in cancer screening before and during the COVID-19 pandemic, annual BRFSS surveys for 2014–2019 were compared to screening percentages for 2020. Since we were interested in changes in screening after the pandemic started, we recoded each of the screening tests into screened anytime less than 12 months ago vs. not within the last 12 months. These rates were examined overall, by race, and by ethnicity to look for disparities. Each month, the BRFSS survey asked about screening in the prior 12 months, so complete impact of the pandemic will take longer to observe. Since the pandemic did not shut down businesses and schools until March 2020, we removed data from January–March 2020 to calculate these screening percentages. Even so, the data for April–December 2020 still reflected some screening conducted prior to the pandemic. Thus, the percentages presented here are conservative.

### 2.3. Analyses

BRFSS data need to be weighted per sampling methods. The weights are provided and described for each BRFSS year on the CDC’s BRFSS website. The data include the core combined landline and cellular telephone survey multiple questionnaire version data, along with three optional modules asked each month of the survey year on both the landline survey and the cellular telephone survey. Thus, each state may use 0–4 different modules to ask the cancer screening questions. The data were included based on which states asked each set of screening questions and which version they asked per the “Complex Sampling Weights and Preparing 2020 BRFSS Module Data for Analysis” (CDC, July 2021). For states in a given year, who collected data via multiple versions of the survey, the sample size for the questions for each version (main dataset and versions 1–3) were summarized across year and state, then the percentage of subjects in each of the four possible versions were calculated (per BRFSS coding instructions). If a state for a given year only used one version, then those data would be weighted as 100%. These percentages were then multiplied by the _FINALWT (BRFSS provided complex sampling weights) to provide the final weights for each version by year and state as per the BRFSS coding manuals. Due to small numbers of data on Native Hawaiian or other Pacific Islander and “other races”, these were not presented unless they classified themselves as multiracial or Hispanic.

Analyses of the BRFSS data accounted for the weighting of survey sampling in SAS using PROC SURVEY MEANS and PROC SURVEYFREQ. Rao–Scott chi-square tests were performed to compare cancer screening rates prior to the pandemic (i.e., 2014–2019) to 2020 for each specific cancer screening, in which cancer screening status was derived based on whether a participant reported screening within the past year as captured in BRFSS data. The screening percentages were compared with an attributable risk (AR, percentage with screening in 2020—percentage with screening in 2014–2019). We can also compare them by using an attributable risk percent (AR%), which divides the AR by percentage with screening in 2020.

## 3. Results

Table 1 describes the BRFSS participants in 2014–2019 compared to 2020 by age, sex, ethnicity, race, obesity, education, employment status, and income. Few differences were seen except for expected increases in 2020 for being out of work for less than one year, slightly more retired participants but fewer homemakers, and more participants at the highest salary category. These factors relate to the stay at home efforts during the beginning of the pandemic.

Reported mammograms in the prior 12 months from the BRFSS survey had a 5.3% reduction in April–December 2020 compared to 2014–2019 for women age 50+, with similar reductions of 7.2% for women ages 40–49, along with an 8.6% reduction in reported pap tests and a reduction in colonoscopy/sigmoidoscopy screening in the last 12 months of 1.3% (Table 2). If we look at the AR% rather than AR, we see an AR% of −8.3, −14.4, and −7.2, respectively, for the age specific screening rates for mammograms (age 50–74), pap tests (ages 21–65), and colonoscopy/sigmoidoscopy screening (ages 50–75) representing reductions in 2020. Screening mammography for women ages 40–49 pre-pandemic were more than 10% lower than among women aged 50–74.

Table 2 reports screening percentages for 2014–2019 and 2020 overall, among Hispanics, and non-Hispanics by racial category. For 2014–2019 and 2020, Hispanics had lower screening percentages for colonoscopy/sigmoidoscopy and mammography among women aged 40–49 than non-Hispanics, but higher screening percentages for mammography among women aged 50–74, and pap tests. The highest screening percentages for mammography and pap tests prior to the pandemic (2014–2019) were among African-Americans/Blacks (Table 2). The lowest screening percentages for pap test for 2014–2019 BRFSS were among Asians, then multiracial participants (Table 2).

Some variation in screening was seen by other factors (data not shown). When looking at reductions in 2020 by age for pap test, younger women (<50) were at the most risk, and women aged 40–49 without college or technical schooling had the highest reduction in screening for mammography. Women with income over $35,000 had more of a reduction in mammography and pap testing, except for mammograms among women aged 40–49 had a higher reduction among those reporting <$25,000. “Homemakers” also had lower screening percentages in 2020 for mammography (40–49 and 50–74), pap tests, and colonoscopy/sigmoidoscopy. Somewhat expected, those out of work for one year or more had high reductions in screening for mammograms and colonoscopy/sigmoidoscopy.

## 4. Discussion

These US BRFSS survey data showed a reduction in cancer screening in 2020, even using our conservative approach that included some 2020 screening reporting received prior to the pandemic (screening in the past 12 months asked April–December 2020). Reductions were seen overall. When screening percentage differences between 2020 and 2014–2019 were stratified by either racial or ethnic groups, all groups saw a reduction, but some were not significant. The AR% for 2020 screening in the prior 12 months among Hispanics showed decreases of 20.5% for mammography in those aged 50–74, 34.7% for pap testing, and 21.1% for colonoscopy/sigmoidoscopy. Participants in the BRFSS self-identifying as Asians had the largest decrease in mammography screening followed by American Indian or Alaskan Natives. For pap tests, the largest drops were seen among Hispanics followed by Whites and Native Hawaiian/Pacific Islanders. The conservative estimates presented here reflect an issue of missed screening that may lead to later stage at diagnosis impacting prognosis. Several studies have shown higher reported screening proportions in the BRFSS survey than the National Health Interview Survey (NHIS) [17,18,19], possibly suggesting that the BRFSS may overestimate the proportion of the population screened. However, when comparing BRFSS data for 2020 to prior years, we were able to look at and see decreases due to the beginning of the pandemic.

Overall screening percentages in the past 12 months for 2014–19 were higher than might be expected for mammograms in those aged 50–74 (61%, recommend biannually starting at age 50), pap test (47% among women aged 21–65 years old, recommended every three years), and colonoscopy/sigmoidoscopy (16%, aged 50–75, recommended every 10 years) based on recommendations that if followed would expect 50%, 33%, and 10%, respectively, each year. Similarly, high rates have been seen over time in the BRFSS survey and the NHIS including higher screening rates in African-Americans/Blacks than Whites [12,17,18,19]. Similar to the lower rates we saw among Asian-Americans even pre-pandemic, others studies have reported lower cancer screening rates in Asian-Americans [20,21]. We saw that the 2020 screening for the prior 12 months overall dropped, even though they were still within the expected ranges based on U.S. Preventive Services Task Force (USPSTF) recommendations [12]. However, cancer screening rates dropped below recommended levels for some racial groups. The larger concerns are that these pandemic estimates are conservative as they include screening prior to the pandemic, with much higher drops likely in 2021.

The Medicaid expansion has seen higher screening rates for cervical and colorectal cancer for low-income adults [13,14]. This may account for higher screening rates in African-Americans and Hispanics seen pre-pandemic along with suggested higher rates of colonoscopy or sigmoidoscopy in American Indians and multiracial participants (Table 2). CRC screening evaluation and analysis is complex due to the multiple modalities for screening offered. With the inclusion of a multitarget stool DNA test in 2016, the number of patients screened with fecal-stool testing (FOBT, FIT, or Cologuard) significantly increased in multiple studies [22,23]. We should note that when surveying participants who self-classified as American Indian/Alaskan Native, they may not represent all Native Americans, particularly those living on Native American lands. The proportion of inadequate cancer screening is higher in American Indians/Alaska Natives [24,25,26].

Some of the reduction in screening in 2020 was likely due to canceled or postponed screening along with a reduction in the patients’ willingness to undergo screening due to the fear of contracting COVID-19 at the doctor’s office or hospital. In some areas, mixed messages were given early in the pandemic. Interpretations of screening recommendations during the pandemic varied from the CDC, WHO, and varied state policies led to nonuniform disruptions in patient care for a wide range of cancer patients and screening, in some cases varying within the same large cities [15,16]. COVID-19 is having an immense impact on cancer screening, diagnosis, prognosis, and treatment [15,16,27] with unclear impact on cancer stage and future cancer survival. In addition to other delays, a COVID-19 diagnosis during cancer treatment stops cancer therapy [28]. Changes in daily activities due to COVID-19 may be reflected in the reduction in percentage of homemakers completing the 2020 BRFSS due to having children at home full-time; and the increase in BRFSS participants of the highest income category may be due to more people working from home in the U.S. during the pandemic.

It is known that medically underserved populations including racial/ethnic minorities exhibit lower uptake of cancer screening than non-minorities [9,12,29,30]. Various medically underserved populations also have disparities in cancer outcomes [31]. Fear of contracting COVID-19 along with structural barriers including limited access to clinics, financial, employment, and transportation issues are concerns that are intensified in medically underserved communities [9]. These issues are likely to create greater disparities. Financial toxicity in the U.S. among cancer patients may range as high as 39–64%, however, it is only 7–39% in publicly funded health care systems that target underserved populations [32,33,34]. Lower financial toxicity for those using publicly funded health care systems may help prevent some increases in disparities due to COVID-19. Nevertheless, employment provides some financial security and often health insurance, so disproportionate pandemic job losses could expand disparities. As the 2020 BRFSS data are conservative estimates, it is unclear of the extent of disparities seen in screening. However, any reduced screening is likely to increase the stage at diagnosis and mortality rates due to these cancers, thus increasing structural inequalities [9]. If the 2021 screening rates drop even more than 2020, as they are expected to, screening rates will be below the recommended rates. Then, the likelihood of catching up to baseline screening rates while, at the same time, not increasing stage at diagnosis or disparities, is particularly concerning [35,36].

Primary prevention is the main strategy to reduce the growing burden of colon, breast, and prostate cancer [37]. With regard to breast cancer alone, national estimates project that the COVID-19 pandemic is leading to an estimated deficit of 3.9 million breast cancer (BC) screenings among U.S. adults [38]. Unfortunately, the reduced rate of screening impacts cancer incidence, treatment, cancer-related mortality, and overall cost of cancer care long-term. Patients who could have been diagnosed at an earlier stage cancer are being diagnosed at more advanced stages. Patients who were directly impacted by COVID-19 are less likely to be screened due to the long-term impact of COVID-19 as well as the psychological stress of disease [39]. With a growing deficit in the screening rates, it has been projected that the COVID-19 pandemic could potentially inflate the mortality from breast cancer and colorectal cancers over the next 10 years by at least 1%, which would equate to 10,000 additional deaths on top of the one million projected deaths from these two cancers alone [39]. The biggest peak of mortality from cancer-related deaths through the COVID-19 pandemic is expected in 2022–2023.

Due to the pandemic, cancer patients were at risk of disruption of treatment at medical facilities [15,40], particularly when hospitals were burdened with high volumes of COVID-19 patients [28,41]. Some facilities revised chemotherapy protocols to minimize both the frequency of chemotherapy visits and the degree of immunosuppression [42]. Two studies, one of radiation oncologists in India and another of medical oncologists in Italy, found that while they may have felt safe, they were still worried about patient safety due to COVID-19 and had some fear of contracting COVID-19 and infecting their families [43,44]. Patients also had similar fears. A study surveyed lung cancer patients who reported anxiety with reasons for anxiety as fear of delaying testing, and fear of contracting the virus, and found a higher than normal percentage of patients who needed referrals to some level of mental health services [45]. Breast cancer patients experienced fear of increased risk of COVID-19 exposure, but an Italian study found that family resilience, coping flexibility, and feeling in control contributed significantly in managing their cancer [40].

The economic impact of delayed cancer screening and the diagnosis of later stage disease over the next 10 years is just as staggering. This impact is likely to create more structural inequalities due to job loss and financial burden. Cancer exerts a significant economic burden on the U.S. health care system [46,47]. This will increase as more people are diagnosed with later stage cancers. Multiple studies have shown that financial toxicity and the economic impact of cancer varies by stage. Costs are nine times more expensive for patients with advanced cancer when compared to patients with early stage cancer [48]. When diagnosed at a later stage, cancer care costs are reported to be higher at the end of life phase when compared to the initial year of cancer treatment [48]. This makes cancer patients and survivors during the pandemic a vulnerable population disproportionately affected by financial burdens since they already spend more out-of-pocket for medical care than patients with other chronic illnesses [49]. With the majority of health insurance in America provided by employers, a loss of a job equates to loss of health insurance. Unemployment increased from 3.8% in February 2020 to 11.2% of Americans in June who were laid off or lost their job secondary to the pandemic with higher rates of unemployment among Blacks (15.1%), Latinos (14.4%), and Asians (13.5%), then Whites (9.2%), with disproportionate losses likely leading to more disparities [50]. Due to the pandemic, many people have lost or are at risk of losing their health insurance [51]. While expanded Medicaid programs appear to have started to close screening gaps among disadvantaged populations, BRFSS data showed a 26–39% lower screening prevalence in woman who lacked health insurance, so pandemic job and insurance losses are concerning [12]. With the loss of insurance, patients may experience a delay in diagnosis (resulting in later stage disease long-term) due to significant delays in screening, delays/breaks in treatment of current cancer patients, or lack of stage appropriate treatment. Cancer survivors with low income at baseline, loss of wages, or perceived social isolation already face higher levels of financial toxicity than their counterparts [52,53]. These are all factors that may be exacerbated by necessary public health measures such as physical distancing throughout the COVID-19 pandemic, creating a large setback in previous reductions in disparities for cancer screening and mortality.

These disparities need to be addressed with community engagement. A systemic review of economic evaluations of interventions leveraging social determinants of health to improve breast, cervical, and colorectal cancer screening found that they appear to be cost-effective for underserved, disadvantaged populations in the United States [54]. Community residents’ and leaders’ engagement as equal partners in structural and systemic interventions is a must for cancer screening programs to address root causes of disparities [55,56]. Future research into collaborative interventions to advance equality would involve those most impacted and aim for sustained community change and transformative change in power, equity, and justice [56].

The strengths of this study include the sample size and inclusion of random samples across all of the US. Additionally, the BRFSS survey includes various underserved populations. Limitations may include a difference in sampling that could explain the higher proportion of mammography screening and pap tests among African-American/Blacks seldom seen elsewhere, but also seen in the NHIS survey. Additionally, reporting of screening may suffer from reporting bias, as do all study designs, which could include over-reporting of screening close to 12 months prior but more than 12 months prior. This could explain the higher cancer screening found in the BRFSS and NHIS surveys. However, such reporting bias should not differ among participants surveyed in 2020 and before, so would have limited effect on these analyses when looking at decreases in screening by racial/ethnic groups. Questions regarding colonoscopy/sigmoidoscopy screening were asked differently in 2020, which was easy to make comparable, but doing so may have created a bias so the CRC analyses presented should be interpreted with care.

## 5. Conclusions

These conservative data, as they included screening in the past 12 months that would have occurred in 2019, suggest larger drops in screening will be seen in 2021 and beyond due to the COVID-19 pandemic. Such decreases in cancer screening in the U.S. are likely to disproportionately affect Hispanics and some other underserved populations more than non-Hispanic White populations. Health care systems need to prepare to try to catch up in some ways with cancer screening. Furthermore, it will take time, money, and re-education for the post-COVID world to return to the pre-pandemic cancer screening recommended rates, particularly in those population groups that were impacted the most.

## Figures and Tables

**Table 1:** Demographic characteristics within the Behavioral Risk Factor Surveillance System in the United States comparing pre-COVID screening (2014–2019) to 2020.

	2014–2019 Weighted % ^a^	2020 Weighted % ^a^
Age		
18 to 39	38.0%	37.9%
40 to 49	16.0%	15.7%
50 to 59	17.5%	16.6%
60 to 69	15.0%	15.3%
70 to 79	9.1%	10.0%
80 or older	4.4%	4.4%
Sex		
Female	51.3%	51.4%
Male	48.7%	48.6%
Hispanic, Latino/a, or Spanish origin		
Not of Hispanic, Latino/a, or Spanish origin	83.1%	81.7%
Don’t know/Refused	1.1%	1.3%
Racial groups		
White	73.0%	70.4%
African American/Black	12.7%	12.9%
American Indian/Alaskan Native	1.9%	1.8%
Asian	5.4%	6.0%
Native Hawaiian/Pacific Islander	0.4%	0.3%
Other	6.7%	8.6%
Obesity based on BMI		
Under or Normal Weight	34.8%	32.9%
Overweight	35.4%	35.0%
Obese	29.8%	32.1%
Education		
Did not graduate High School	13.7%	12.1%
Graduated High School	27.9%	27.3%
Attended College or Technical School	30.9%	30.6%
Graduated from College or Technical School	26.9%	29.4%
Don’t know/Refused	0.6%	0.5%
Own or Rent Your Home		
Own	66.1%	65.8%
Rent	27.2%	26.8%
Other arrangement	5.7%	6.3%
Don’t know/Refused	1.0%	1.1%
Employment		
Employed for wages	47.6%	46.5%
Self-employed	8.8%	8.7%
Out of work for 1 year or more	2.7%	2.4%
Out of work for less than 1 year	2.9%	6.2%
A homemaker	6.3%	4.8%
A student	5.6%	5.1%
Retired	18.0%	18.8%
Unable to work	6.9%	6.3%
Refused	1.0%	1.2%
Salary		
Less than $15,000	9.2%	7.4%
$15,000 to less than $25,000	14.1%	12.0%
$25,000 to less than $50,000	20.0%	18.0%
$50,000 to less than $75,000	12.6%	12.5%
$75,000 or more	28.0%	31.5%
Don’t know/Refused	16.1%	18.7%

^a^ Percentage based on weighted frequencies.

**Table 2:** Had a cancer screening test in the last 12 months based on the Behavioral Risk Factor Surveillance System in the United States comparing pre-COVID screening (2014–2019) to 2020 across racial/ethnic groups ^a^.

		2014–2019			2020 ^b^				
Population	Cancer Screening Tool	Frequency	Weighted Frequency	Percent ^c^	Frequency	Weighted Frequency	Percent ^c^	ChiSq	AR ^d^
	Had a mammogram (aged 50–74)								
All subjects	Yes	235,210	64,177,218	61.4%	51,891	19,706,934	56.2%	<0.0001	−5.3%
	No	148,620	40,277,837		39,362	15,374,965			
Hispanic	Yes	10,448	5,964,793	61.1%	2476	2,114,660	51.9%	<0.0001	−9.2%
	No	7120	3,799,483		2174	1,961,598			
Non-Hispanic:									
African American or Black only	Yes	20,070	8,016,487	67.0%	4539	2,605,698	63.8%	0.019	−3.2%
	No	9515	3,945,688		2732	1,478,414			
American Indian or Alaskan Native only	Yes	2796	613,660	58.6%	652	158,921	46.3%	0.0006	−12.3%
	No	2305	434,000		777	184,462			
Asian Only	Yes	2813	2,168,454	62.9%	703	536,062	41.7%	<0.0001	−21.2%
	No	1589	1,279,180		543	749,920			
Multiracial	Yes	3431	638,893	52.4%	755	174,336	46.1%	0.0683	−6.3%
	No	2742	580,548		692	203,508			
White only	Yes	192,349	45,871,458	60.8%	41,638	13,745,834	56.9%	<0.0001	−4.0%
	No	122,726	29,557,765		31,451	10,430,263			
	Had a mammogram (aged 40–49)								
All subjects	Yes	45,327	20,777,085	48.5%	10,763	5,705,663	41.3%	<0.0001	−7.2%
	No	47,094	22,032,813		14,314	8,093,537			
Hispanic	Yes	4432	3,259,739	45.5%	1133	952,735	34.8%	<0.0001	−10.7%
	No	5390	3,902,908		1874	1,783,253			
Non-Hispanic:									
African American or Black only	Yes	4581	3,078,799	53.5%	1219	1,020,685	49.1%	0.0313	−4.3%
	No	3763	2,679,324		1359	1,056,193			
American Indian or Alaskan Native only	Yes	731	2,188,77	47.2%	169	53,624	35.4%	0.0365	−11.9%
	No	949	244,396		301	97,956			
Asian Only	Yes	1043	1,119,362	48.2%	294	257,813	34.7%	0.0013	−13.5%
	No	1144	1,202,138		440	484,676			
Multiracial	Yes	917	272,585	43.8%	210	68,631	31.8%	0.0151	−12.0%
	No	1027	349,076		377	147,196			
White only	Yes	32,923	12,501,620	48.6%	7426	3,210,653	42.6%	<0.0001	−6.0%
	No	33,931	13,221,289		9523	4,329,853			
	Had a pap test (aged 21–65)								
All subjects	Yes	211,901	99,656,028	46.6%	45,114	26,735,450	38.0%	<0.0001	−8.6%
	No	261,459	114,302,528		76,526	43,650,981			
Hispanic	Yes	20,028	16,333,829	47.3%	4866	4,758,782	36.0%	<0.0001	−11.3%
	No	22,308	18,194,952		7920	8,442,500			
Non-Hispanic:									
African American or Black only	Yes	22,528	15,241,219	53.9%	5087	4,401,477	48.1%	<0.0001	−5.7%
	No	19,268	13,043,618		5888	4,742,189			
American Indian or Alaskan Native only	Yes	3276	1,037,872	44.9%	788	294,711	40.1%	0.0922	−4.8%
	No	4622	1,275,659		1486	440,598			
Asian Only	Yes	4012	4,019,124	36.2%	1152	1,353,019	29.9%	0.001	−6.3%
	No	5897	7,087,754		2293	3,176,931			
Multiracial	Yes	4432	1,509,572	43.3%	1138	426,418	37.8%	0.0145	−5.4%
	No	5682	1,980,293		1796	700,300			
White only	Yes	154,262	60,020,454	45.9%	30,900	14,899,590	37.0%	<0.0001	−8.8%
	No	199,295	70,839,396		55,280	25,327,282			
	Had a colonoscopy or sigmoidoscopy (aged 50–75)								
All subjects	Yes	116,548	34,365,127	16.3%	19,235	8,437,256	15.0%	<0.0001	−1.3%
	No	611,222	176,967,964		113,089	47,821,992			
Hispanic	Yes	4788	2,977,112	15.0%	835	789,032	12.7%	0.0575	−2.3%
	No	27,136	16,833,066		5939	5,427,495			
Non-Hispanic:									
African American or Black only	Yes	10,007	4,516,440	19.6%	1789	1,301,824	19.6%	0.9796	0.0%
	No	40,133	18,536,405		8098	5,334,547			
American Indian or Alaskan Native only	Yes	1494	394,105	17.8%	304	82,345	13.8%	0.0698	−4.1%
	No	7965	1,819,625		1922	516,422			
Asian Only	Yes	1245	901,049	12.6%	251	183,020	10.6%	0.2192	−2.0%
	No	7891	6,228,146		1805	1,544,568			
Multiracial	Yes	1927	402,732	16.5%	335	73,575	12.7%	0.0117	−3.8%
	No	10,043	2,036,683		1919	503,519			
White only	Yes	95,078	24,643,361	16.1%	15,261	5,850,734	14.9%	<0.0001	−1.2%
	No	507,004	128,410,286		90,377	33,369,472			

^a^ Subjects who checked did not know, refused, or otherwise missing for whether or not they were screening with a specific screening test were excluded from that screening test. Surveys from Guam, Puerto Rico, and the Virgin Island were excluded. ^b^ Among surveys from April–December 2020, which still includes non-pandemic months as a conservative estimate. ^c^ Percent = weighted frequency for “yes” over the weighted frequencies for yes and no, so N = yes + no. ^d^ AR = percentage with screening in 2020—percentage with screening in 2014–2019.

## Data Availability

The data presented in this study are openly available through https://www.cdc.gov/brfss/index.html (accessed on 2 September 2021) and are available from the CDC.
